# Cryo-EM structure of the TL1A-DR3 complex and implications for the treatment of Inflammatory Bowel Disease

**DOI:** 10.1063/4.0000819

**Published:** 2025-10-27

**Authors:** Cameron L Noland, Marcelo Murai, Paulo Zaragoza, Ben Bell, Sultan Yilmaz, Shruti Nayak, Esme Alarcon, Michael Eddins, Todd Mayhood, Yunpeng Zhou, Burt Barnett, Haihong Zhou, Johan Fransson

**Affiliations:** Merck & Co., Inc., Rahway, NJ, USA

## Abstract

TNF-like ligand 1A (TL1A) is a pro-inflammatory cytokine that is a critical regulator of mucosal immunity and has been implicated in the pathogenesis of Inflammatory Bowel Disease (IBD). TL1A is expressed on antigen-presenting cells and binds to its functional receptor, death- domain receptor 3 (DR3), on T cells to activate pro-inflammatory pathways that are integral to the adaptive immune response. In contrast, binding to soluble decoy receptor 3 (DcR3) prevents pathway signaling through direct competition with DR3. TL1A is upregulated in inflamed IBD tissue, and blocking TL1A has shown promise in Ph2 ulcerative colitis and Crohn’s disease studies. However, the structural understanding of the TL1A- DR3 interaction is incomplete, as only the structure of TL1A bound to DcR3 has been elucidated. To gain insights into the molecular nature of the TL1A-DR3 interaction, we solved the structure of this complex to 2.8 Å resolution using cryo-electron microscopy. The structure reveals that DR3 engages TL1A in a manner akin to DcR3, but with several distinct features. Overall, this work deepens our understanding of TL1A-DR3 interactions and lends valuable insights to the development of TL1A inhibitors as a potential treatment for IBD.

